# Multidimensional analysis of childbirth experiences on Chinese social media: an integrated approach using LDA, sentiment analysis and deep learning

**DOI:** 10.3389/fpubh.2026.1788925

**Published:** 2026-04-20

**Authors:** Luping Wu, Wen Bao, Suwen Feng

**Affiliations:** 1 Department of Obstetrics, Women’s Hospital, School of Medicine, Zhejiang University, Hangzhou, China; 2 Information Center, Women’s Hospital, School of Medicine, Zhejiang University, Hangzhou, China; 3 Department of Nursing, Women’s Hospital, School of Medicine, Zhejiang University, Hangzhou, China

**Keywords:** childbirth experience, deep learning, natural language processing, sentiment analysis, social media analysis, topic modeling

## Abstract

**Objective:**

Childbirth is a major life event with complex physical and psychosocial consequences. This study examined how women’s childbirth experiences are constructed in Chinese social media narratives and identified key themes and emotional patterns across different modes of delivery.

**Methods:**

We conducted a cross-sectional content analysis of 17,783 childbirth-related posts from Weibo and REDnote. Latent Dirichlet allocation (LDA) was used to extract topics, and a pre-trained Chinese BERT model was fine-tuned for three-class, sentence-level sentiment classification; model performance was evaluated using accuracy, F1-score and AUC-ROC.

**Results:**

LDA identified six main themes, with “vaginal birth process and pain experience” and “emotional meanings and family value of childbirth” accounting for the largest proportions. Negative emotions substantially outweighed positive ones and were particularly concentrated in vaginal birth narratives, whereas caesarean section and postpartum recovery posts were more neutral and informational. The integrated LDA–BERT approach achieved good performance (AUC-ROC = 0.89), supporting the robustness of sentiment estimates.

**Conclusion:**

Childbirth narratives on Chinese social media present a predominantly negative yet “bittersweet” emotional profile, with marked differences between vaginal and caesarean experiences. These findings underscore the need for tailored perinatal interventions, including individualized labour pain management, strengthened psychological support and more nuanced online health communication.

## 1 Introduction

Childbirth is a major life event for women that involves not only complex physiological processes but also profound psychological and social consequences (1–3). In recent years, with the rapid diffusion of mobile Internet and social media in China, an increasing number of women have started to share their childbirth stories, seek emotional support and exchange medical information on platforms such as Weibo and REDnote. Studies show that Chinese expectant mothers widely use social media and pregnancy-related apps to search for delivery and postpartum information and to communicate with peers (4), while similar patterns of social support seeking have been documented in other countries (5). These user-generated narratives constitute a valuable data source for understanding women’s perceptions, emotions and needs around childbirth from their own perspective.

Traditional research on childbirth attitudes and experiences mainly relies on questionnaires, in-depth interviews and clinical observation. Systematic reviews indicate that such studies have identified important predictors and outcomes of childbirth experience, but are often constrained by sample size, timeliness and representativeness, and tend to emphasize medical outcomes over women’s holistic perceptions of the childbirth process (2, 6). At the same time, highly emotional or fragmented narratives on social media may amplify negative experiences and reinforce fear of childbirth, thereby influencing pregnant women’s expectations and mental health (4, 6). Systematically analysing childbirth narratives on social media is therefore essential for capturing women’s authentic concerns and emotional trajectories, correcting potential cognitive bias and improving perinatal care.

Advances in natural language processing (NLP) now make it possible to process large-scale unstructured text and extract latent patterns efficiently. Latent Dirichlet allocation (LDA) is a widely used unsupervised topic model that represents each document as a mixture of topics and each topic as a distribution over words, and has been successfully applied to online health communities and public-health–related social media data to uncover patients’ concerns and disease-related themes (7, 8). In parallel, deep learning models such as BERT have markedly improved sentiment-analysis performance on health-related social media by learning contextualised representations from large-scale pre-training (8, 9). However, existing health communication studies seldom integrate LDA topic modeling with BERT-based sentiment analysis to examine childbirth experiences in a unified framework, and systematic evidence based on Chinese social media platforms remains scarce (4).

Against this backdrop, this study aims to conduct a multidimensional analysis of women’s childbirth experiences on Chinese social media. We collect and clean more than 17,000 childbirth-related posts from Weibo and REDnote, apply LDA to identify core narrative themes, and use a fine-tuned BERT model to perform sentence-level sentiment classification. Model performance is evaluated using accuracy, F1-score and AUC-ROC. By combining topic structures with sentiment distributions, we seek to reveal women’s main concerns, emotional patterns and underlying needs during the childbirth process, and to provide empirical evidence for improving clinical practice and public-health strategies.

## 2 Related work

### 2.1 Childbirth experiences and maternal well-being

Childbirth is a major life event with profound implications for women’s physical and psychological well-being. A large body of work shows that the subjective experience of birth—not only clinical indicators such as mode of delivery—strongly predicts postpartum mental health outcomes, including depressive symptoms, anxiety, and childbirth-related post-traumatic stress disorder (CB-PTSD) (10). Systematic reviews suggest that between 3 and 4% of women meet diagnostic criteria for CB-PTSD, while a much larger proportion report clinically relevant traumatic stress responses following birth (11, 12).

Research has also emphasized that traumatic or highly negative birth experiences can have long-term psychosocial consequences, including impaired bonding with the infant, fear of future childbirth, and avoidance of subsequent pregnancies (10, 13). Qualitative meta-syntheses describe how women who perceive childbirth as traumatic often struggle with intense feelings of fear, loss of control, and violation, which may persist for years and disrupt identity and relationships (13, 14).

In response, policy and clinical guidelines have increasingly stressed that a “positive childbirth experience” should be a core goal of maternity care, rather than focusing solely on survival and physical safety. The World Health Organization, for example, defines a positive childbirth experience as one that meets or exceeds a woman’s prior expectations and supports her sense of agency, dignity, and emotional security (15). Recent concept analyses similarly argue that psychological birth trauma needs to be understood from women’s own appraisals of threat, violation, and loss of control, and call for more nuanced tools to capture these subjective dimensions (16, 17).

Most of this literature, however, draws on structured questionnaires or interviews conducted in clinical or community settings. While these methods are essential, they may under-represent women who do not access formal services or who feel unable to disclose highly negative experiences in face-to-face encounters. This gap has motivated growing interest in more naturalistic, large-scale data sources such as online narratives.

### 2.2 Childbirth narratives and online social media

Social media platforms have become important spaces where women document pregnancy, labor, and postpartum recovery, seek peer support, and negotiate norms around “good” motherhood (18). Compared with traditional surveys, user-generated content is more spontaneous and less constrained by researcher-defined questions, making it a valuable window into how childbirth is experienced, remembered, and publicly narrated over time.

Studies using forums and Reddit communities have shown that pregnant women and new mothers frequently use online spaces to express worries about pregnancy complications, delivery pain, and neonatal health, as well as to exchange practical tips and emotional support (19, 20). Topic-modeling analyses of pregnancy-related subreddits indicate that conversations revolve around both medical risk (e.g., preeclampsia, fetal growth, induction) and everyday stressors (e.g., body image, family conflict, work–life balance) (19, 20).

In the Chinese context, Sina Weibo and other social media platforms have been widely used to examine public discourses on depression and mental health, revealing how stigma, help-seeking, and coping are negotiated in online environments (19, 20). Large-scale analyses of Weibo posts have shown that linguistic and topical patterns can capture users’ emotional states and perceived stressors in ways that complement traditional epidemiological data (21, 22).

However, empirical work focusing specifically on Chinese social-media childbirth narratives remains limited. Existing studies often treat pregnancy and postpartum mental health at a relatively aggregate level (e.g., “perinatal depression” or “maternal stress”), rather than examining how distinct dimensions of the birth experience—such as mode of delivery, intrapartum care interactions, and postpartum recovery—are thematized and emotionally evaluated in women’s own narratives. There is also a lack of fine-grained comparison between vaginal birth and caesarean section stories in everyday online talk.

### 2.3 NLP and deep learning for maternal mental health on social media

Parallel to these developments, computational methods have been increasingly applied to social-media data to detect depression, anxiety, and other mental-health conditions. Early work showed that linguistic markers and posting behaviors on platforms like Twitter and Facebook can predict clinical depression and post-traumatic stress disorder with promising accuracy (23, 24). Subsequent studies extended these approaches to perinatal populations, using machine learning to identify postpartum depression risk and characterize its linguistic signatures (25).

In the Chinese social-media context, transformer-based natural language processing (NLP) models have been developed to predict depression and suicidal ideation from large-scale Weibo corpora, demonstrating that contextualized language representations (e.g., BERT-style models) substantially outperform earlier lexicon-based or shallow machine-learning approaches (22, 26). A recent scoping review on AI for postpartum depression similarly concludes that deep-learning models, especially those leveraging text and social-media traces, hold considerable promise but remain under-applied in perinatal mental-health research compared with general depression detection (27).

At the same time, there is a methodological shift from coarse binary classification (e.g., depressed vs. non-depressed) toward topic-aware sentiment and emotion analysis, which jointly models what people are talking about and how they feel about those topics (22, 28). This line of work is particularly relevant for childbirth, where narratives often intertwine pain, fear, relief, and joy in complex ways. Yet existing computational studies seldom zoom in on childbirth itself as a distinct analytic focus, nor do they systematically compare sentiment patterns between vaginal and caesarean birth stories within the same cultural and platform context.

Against this backdrop, the present study contributes by (1) focusing specifically on childbirth narratives in Chinese social media; (2) combining topic modeling with a BERT-based sentiment classifier tailored to childbirth-related content; and (3) contrasting the thematic and emotional profiles of vaginal versus caesarean birth posts. In doing so, it bridges clinical research on childbirth experiences with computational social-media analytics and extends prior work on perinatal mental health into the under-explored domain of everyday online birth storytelling.

## 3 Methodology

### 3.1 Data collection and preprocessing

**Figure 1:**
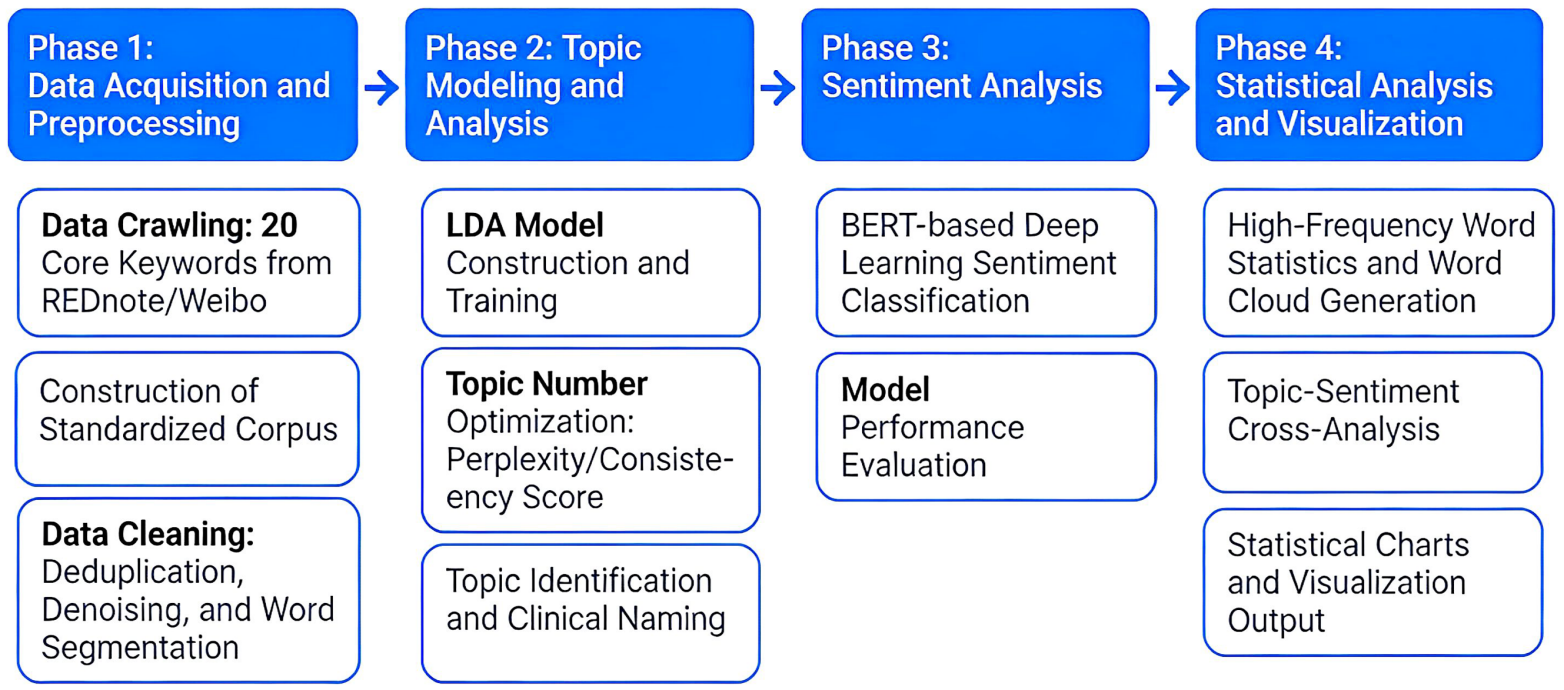
Four-stage analytical workflow for social media childbirth narrative analysis.

The four-stage analytical workflow of this study is illustrated in Figure 1. This study adopted a retrospective design and used Python (version 3.7) to develop a web-crawling script. Childbirth-related posts published between October 1, 2023, and September 30, 2025, were collected from two major Chinese social media platforms, Weibo and REDnote. To capture user–generated narratives of childbirth experiences as comprehensively as possible, a set of keyword combinations was constructed around delivery modes and experience-sharing expressions (e.g., “vaginal birth diary,” “caesarean section diary,” “childbirth story,” “childbirth experience sharing”). Posts matching these keywords within the pre-defined time window were retrieved and stored.

In the preprocessing stage, regular expressions were used to remove emojis, URLs, punctuation and other non-Chinese characters, thereby reducing textual noise. The remaining text was then segmented using the Jieba tokenizer. To improve tokenization accuracy for obstetric content, a domain-specific medical lexicon was added, including terms such as “epidural analgesia,” “induction of labour” and “oxytocin.” After segmentation, a customized stop-word list (e.g., function words such as “的,” “了,” “在”) was applied, and only meaningful tokens with a length greater than one character were retained for subsequent analysis.

### 3.2 High-frequency word analysis

**Figure 2:**
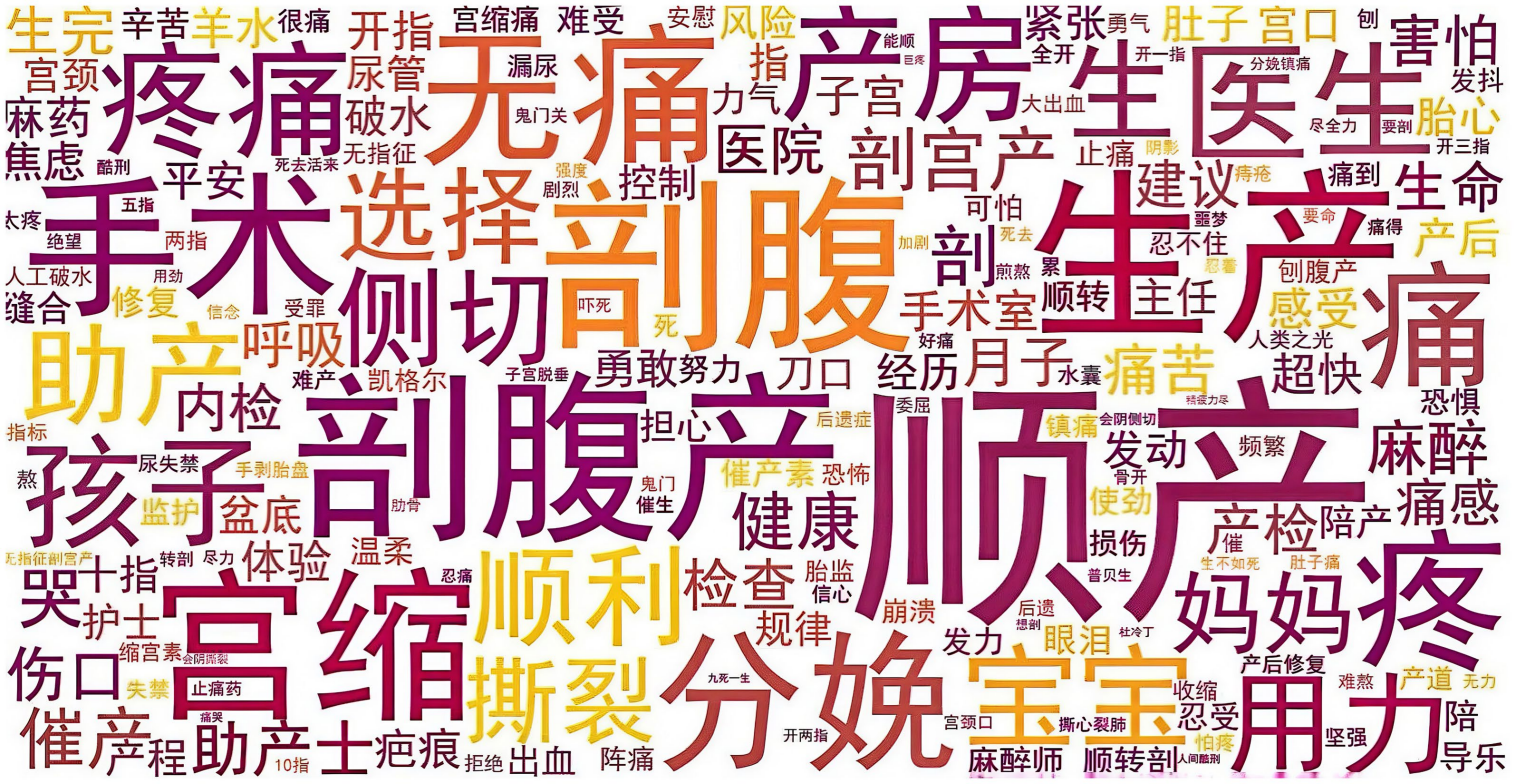
Word cloud of themes about childbirth.

Following preprocessing, word frequency statistics were computed for all valid tokens to obtain a macro-level view of the most salient concepts discussed in the corpus. Using Python’s collections library, term frequencies were counted and sorted in descending order. The top 100 high-frequency words were then examined to identify the core semantic foci of childbirth discussions and to provide preliminary support for the topic structures revealed by the subsequent LDA modeling. The word cloud of themes about childbirth is drawn in Figure 2. To ensure accessibility for readers who do not read Chinese, the high-frequency terms presented in the word cloud are listed in Table 1 along with their English translations and frequencies.

**Table 1:** High-frequency terms with English translations and frequencies.

No.	Chinese	English	Count
1	顺产	Natural birth	14,335
2	剖腹	C-section	5,910
3	生产	Giving birth	5,858
4	剖腹产	C-section	5,596
5	宫缩	Contractions	4,405
6	手术	Surgery	4,149
7	分娩	Delivery	3,894
8	疼	Hurt	3,876
9	无痛	Painless	3,850
10	痛	Pain	3,098
11	产房	Delivery room	2,658
12	疼痛	Pain	2,590
13	助产	Birth assistance	2098
14	用力	Push	2041
15	侧切	Cut	1986
16	顺利	Smooth	1960
17	选择	Choice	1947
18	撕裂	Tear	1919
19	剖宫产	C-section	1753
20	剖	C-section	1,683
21	催产	Induced	1,678
22	哭	Cry	1,651
23	健康	Healthy	1,561
24	助产士	Midwife	1,535
25	麻醉	Epidural	1,511
26	伤口	Incision	1,496
27	检查	Check-up	1,361
28	痛苦	Agony	1,337
29	产检	Prenatal visits	1,331
30	月子	Postpartum month	1,313
31	害怕	Scared	1,275
32	内检	Internal check	1,250
33	生命	Life	1,248
34	生完	After birth	1,245
35	建议	advice	1,139
36	痛感	pain level	1,103
37	呼吸	Breathing	1,091
38	手术室	OR	1,077
39	超快	Super fast	1,031
40	感受	Feeling	1,018
41	麻药	Meds	1,015
42	子宫	Uterus	1,006
43	体验	Experience	1,005
44	羊水	Water	920
45	开指	Dilated	909
46	破水	Water broke	902
47	疤痕	Scar	874
48	平安	Safe and sound	847
49	发动	Labor started	837
50	胎心	Baby heartbeat	835
51	盆底	Pelvic floor	825
52	主任	Chief doctor	819
53	焦虑	Anxious	809
54	紧张	Nervous	806
55	宫口	Cervix	795
56	指	cm	795
57	勇敢	Brave	772
58	十指	Fully dilated	764
59	产程	Labor process	764
60	控制	Control	724
61	刀口	Scar	716
62	尿管	Catheter	705
63	风险	Risk	681
64	规律	Regular	679
65	宫颈	Cervix	671
66	陪产	Birth partner	633
67	担心	Worried	596
68	缝合	Stitched up	596
69	力气	Strength	566
70	难受	Miserable	550
71	出血	Bleeding	547
72	修复	Recovery	541
73	顺转	Switched to C-section	532
74	陪	Accompany	519
75	眼泪	Tears	512
76	辛苦	Hard work	510
77	导乐	Doula	493
78	顺转剖	Failed vaginal birth turned C-section	486
79	催产素	Pitocin	475
80	麻醉师	Anesthesiologist	469
81	努力	Trying hard	460
82	温柔	Gentle	456
83	使劲	Pushing hard	454
84	忍受	Enduring	452
85	发力	Pushing	427
86	恐惧	Terrified	408
87	止痛	Pain relief	398
88	监护	Monitoring	397
89	可怕	Terrifying	396
90	损伤	Damage	383
91	宫缩痛	Contraction pain	372
92	阵痛	Labor pains	332
93	死	Death	322
94	崩溃	Breaking down	321
95	忍不住	Could not take it	303
96	镇痛	Pain relief	303
97	很痛	Hurts so bad	296
98	刨腹产	C-section	289
99	产道	Birth canal	287
100	发抖	Shaking	286

### 3.3 Topic modeling with LDA

#### 3.3.1 Rationale for method selection

Latent Dirichlet allocation (LDA) was selected as the topic-modeling technique. LDA is an unsupervised probabilistic generative model that automatically identifies latent topic structures from massive texts without predefined labels, making it highly suitable for exploratory research on social media data. Compared to traditional word frequency or clustering methods, LDA reveals the dual probability distributions of document-topic and topic-word, which better reflects the mixed semantic nature of textual narratives.

#### 3.3.2 Model training and optimization

**Figure 3:**
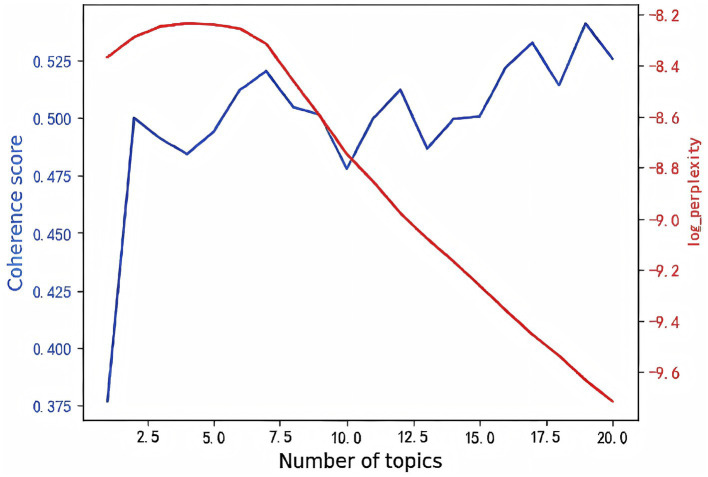
Perplexity and topic coherence scores for determining the optimal number of topics.

To determine an appropriate number of topics, we estimated models with topic numbers ranging from 1 to 20 and compared them using perplexity and topic-coherence scores. Perplexity reflects the uncertainty of assigning documents to topics (lower values indicate better generalization), whereas topic coherence measures the semantic consistency among high-probability words within a topic (higher values indicate better interpretability). As shown in Figure 3, when the number of topics was set to six, the perplexity curve exhibited an elbow (perplexity = −8.37) and the coherence score reached its maximum (coherence = 0.63), suggesting that the six-topic solution achieved an optimal balance between model complexity and interpretability. During model training, the number of iterations was set to 50 and the random seed was fixed at 100 to ensure convergence and reproducibility.

**Figure 4:**
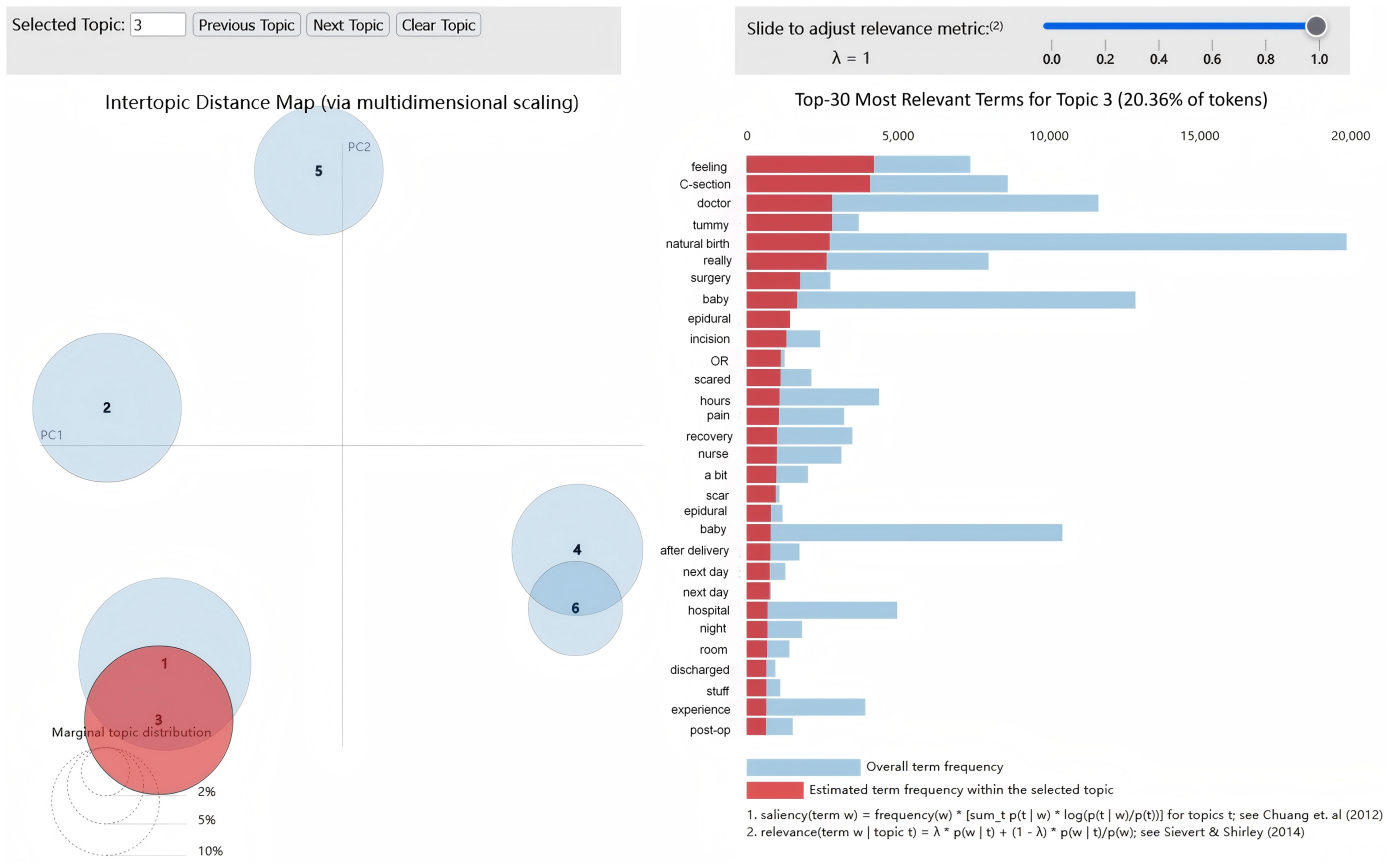
Intertopic distance map and term relevance using pyLDAvis.

For the selected six-topic model, we examined the top-weighted keywords under each topic and conducted manual, clinically informed interpretation of their meanings. On this basis, we assigned descriptive labels and definitions to the six topics. The topic labels and their representative terms (with corresponding weights) are summarized in Table 2. Topic visualisation was implemented using the Python library pyLDAvis, which generates an interactive HTML report of intertopic distances and term relevance; a static screenshot of this visualisation is presented in Figure 4.

**Table 2:** Six topics identified by the lda topic model.

Topic label	Representative keywords and weights
Topic 1 Vaginal birth process and experience (proportion 28.76%)	0.025*"doctor” + 0.019*"uterine contractions” + 0.018*"painless” + 0.014*"vaginal birth” + 0.012*"pushing” + 0.011*"giving birth” + 0.010*"hour” + 0.009*"midwife” + 0.009*"feeling” + 0.009*"tearing”
Topic 2 Emotional value and family meaning of childbirth (proportion 27.2%)	0.032*"child” + 0.027*"mother” + 0.013*"really” + 0.012*"baby” + 0.010*"experience” + 0.009*"vaginal birth” + 0.007*"husband” + 0.006*"pain/suffering” + 0.005*"woman” + 0.005*"life”
Topic 3 Pregnancy diaries and mother–baby experience sharing (proportion 20.36%)	0.051*"vaginal birth” + 0.026*"delivery” + 0.020*"child” + 0.014*"baby” + 0.012*"caesarean section” + 0.012*"labour” + 0.012*"mother” + 0.011*"pregnancy” + 0.010*"pregnancy period” + 0.010*"sharing”
Topic 4 Postpartum recovery (proportion 8.44%)	0.021*"postpartum” + 0.015*"caesarean section” + 0.015*"vaginal birth” + 0.015*"recovery” + 0.010*"scar” + 0.008*"nursing/care” + 0.007*"wound” + 0.006*"body” + 0.006*"condition” + 0.006*"uterus”
Topic 5 Caesarean section surgical experience (proportion 10.85%)	0.020*"feeling” + 0.019*"caesarean section” + 0.013*"doctor” + 0.013*"belly/abdomen” + 0.012*"really” + 0.008*"surgery” + 0.007*"anaesthesia” + 0.006*"wound” + 0.005*"fear” + 0.005*"pain” + 0.003*"really good / ‘so worth it’” + 0.002*"comfortable”
Topic 6 High-risk pregnancy and obstetric emergency care (proportion 4.40%)	0.015*"placenta” + 0.013*"hospital” + 0.012*"doctor” + 0.007*"caesarean section” + 0.006*"amniotic fluid” + 0.005*"delivery” + 0.004*"examination” + 0.003*"fetal position” + 0.003*"umbilical cord” + 0.003*"emergency”

### 3.4 Sentiment analysis with a BERT-based model

#### 3.4.1 Rationale for BERT model

For fine-grained sentiment analysis, a BERT (Bidirectional Encoder Representations from Transformers)-based deep learning model was adopted. Traditional lexicon-based methods struggle with complex linguistic phenomena (e.g., negation, irony) in Chinese social media texts, whereas BERT captures contextual semantics through its bidirectional Transformer architecture. Furthermore, its “pre-training + fine-tuning” paradigm exhibits high sample efficiency, and it significantly outperforms traditional machine learning (e.g., SVM) and RNN-based models (e.g., Bi-LSTM) in Chinese sentiment classification tasks.

#### 3.4.2 Manual annotation process and quality control

To train the BERT sentiment classification model, a subset of 2,000 posts was randomly selected for manual annotation. The annotation team consisted of two graduate students with sociology backgrounds who received systematic training in NLP annotation tasks. They were trained using guidelines with 50 sample posts to define three sentiment labels: positive, neutral, and negative. During the formal annotation phase, the two researchers coded the posts independently. Cross-checking was conducted periodically, and any discrepancies were resolved by a third senior researcher with an obstetric background. The inter-annotator agreement was high, with a Cohen’s Kappa coefficient of 0.85 (95% CI: 0.82–0.88), indicating excellent annotation reliability.

#### 3.4.3 Sample size justification and model performance

The sample size of 2,000 training instances is robust, as previous studies have demonstrated that fine-tuning BERT requires as few as 100 to 500 labeled texts to achieve strong performance in domain-specific classification tasks. During the fine-tuning process, the pre-trained bert-base-chinese model was optimized using AdamW with a learning rate of 2e-5 and a weight decay of 0.01. The maximum sequence length was set to 128, the batch size was 16, and the model was trained for 5 epochs using an early stopping strategy based on validation loss. The fine-tuned BERT model on the test set of 15,783 posts achieved an accuracy of 0.785 (95% CI: 0.76–0.80) and an AUC-ROC of 0.890. To validate its effectiveness, a Bi-LSTM model was constructed as a baseline, which yielded lower performance (accuracy: 0.742; AUC-ROC: 0.852). These results confirm that the BERT model can reliably identify sentiments in Chinese social media texts.

### 3.5 Ethical considerations

All data used in this study were obtained from publicly accessible social media platforms. Before analysis, user-identifiable information was removed and the corpus was fully de-identified. The research protocol was reviewed and approved by the Ethics Committee of the Women’s Hospital, Zhejiang University School of Medicine (Approval No. IRB-20250386-R), and informed consent was waived in accordance with the regulations for secondary analysis of anonymized data.

## 4 Results

### 4.1 Descriptive statistics of the corpus

Using a Python-based crawler, we retrieved childbirth-related posts from Weibo and REDnote After removing duplicates, advertisements, institutional posts and entries without substantive narrative content, more than 17,000 valid posts were retained, with each platform contributing roughly half of the corpus.

Most posts were written by women who had personally experienced childbirth and were sharing their labour process, delivery mode, or postpartum recovery. The average length of a post was around several hundred characters, with some posts being multi-part “diaries” covering the entire process from late pregnancy to early parenting. High-frequency word statistics showed that terms related to mode of delivery (“vaginal birth,” “caesarean section”), labour process and bodily sensations (“contractions,” “pain,” “epidural anaesthesia,” “oxytocin”), and medical and family support (“doctor,” “midwife,” “nurse,” “baby,” “husband”) appeared most frequently, providing an initial indication of the core concerns in the corpus.

### 4.2 Topic structures of childbirth narratives

To uncover latent thematic structures, we applied latent Dirichlet allocation (LDA) to the preprocessed corpus. Models with different topic numbers (1–20) were estimated and compared using perplexity and coherence scores. The six-topic model achieved the lowest perplexity and highest coherence and was therefore selected as the optimal specification.

Clinical interpretation of the high-probability keywords under each topic led to the following themes and proportions:

Theme 1: Vaginal birth process and experience (≈28–29%) – descriptions of uterine contractions, labour stages, pain intensity, epidural analgesia and interactions with doctors and midwives.
Theme 2: Emotional value and family meaning of childbirth (≈27%) – reflections on the value of having a child, changes in family relationships, gratitude and love, and the perceived meaning of motherhood.
Theme 3: Pregnancy diaries and mother–baby experience sharing (≈20%) – serial narratives from pregnancy to childbirth and early parenting, often marked as “diaries” and oriented toward sharing.
Theme 4: Postpartum recovery (≈8–9%) – concerns about postpartum bleeding, wound care, breastfeeding difficulties, physical weakness and rehabilitation.
Theme 5: Caesarean section surgical experience (≈10–11%) – the anaesthesia process, intraoperative sensations, postoperative pain, wound healing and mixed feelings about the operation.
Theme 6: High-risk pregnancy and emergency obstetric interventions (≈4–5%) – focusing on placental problems, abnormal fetal position, emergency surgery and intensive monitoring.

Overall, “vaginal birth process and experience” and “emotional value and family meaning of childbirth” together accounted for more than half of the corpus, indicating a dual focus on the embodied trial of labour and the symbolic transformation of becoming a mother.

### 4.3 Sentiment distribution across topics

A BERT-based deep learning model, fine-tuned on manually annotated data, was used to classify each post into positive, neutral or negative (risk-laden) sentiment. At the corpus level, negative posts clearly outnumbered positive posts, while neutral posts constituted nearly half of all entries, reflecting a mixture of emotionally charged narratives and relatively descriptive, informational content.

As shown in Table 3, sentiment distributions varied substantially across topics. In Theme 1 “Vaginal birth process and experience,” negative posts accounted for the highest proportion, predominantly expressing fear, breakdown and helplessness in response to intense contraction pain, extreme physical exhaustion and uncertainty about the process. Theme 2 “Emotional value and family meaning of childbirth” likewise contained a relatively high share of negative emotions, but at the same time showed the highest proportion of positive content among all topics, reflecting a typical “bittersweet” pattern in childbirth narratives, in which suffering and meaning often coexist.

**Table 3:** Sentiment distribution by topic in childbirth narratives.

Topic name	Total number	Total percentage	Sentiment category	Number	Percentage
Topic 1 Vaginal birth process and experience	5,115	28.76%	Negative	2,234	43.68%
			Neutral	2,133	41.70%
			Positive	748	14.62%
Topic 2 Emotional value and family meaning of childbirth	4,837	27.20%	Negative	2006	41.47%
			Neutral	1816	37.54%
			Positive	1,015	20.98%
Topic 3 Pregnancy diaries and mother–baby experience sharing	3,620	20.36%	Negative	925	25.56%
			Neutral	1798	49.67%
			Positive	897	24.78%
Topic 4 Postpartum recovery	1,500	8.44%	Negative	380	25.33%
			Neutral	1,035	69.00%
			Positive	85	5.67%
Topic 5 Caesarean section surgical experience	1929	10.85%	Negative	299	15.50%
			Neutral	1,345	69.73%
			Positive	285	14.78%
Topic 6 High-risk pregnancy and obstetric emergency care	782	4.40%	Negative	274	35.04%
			Neutral	417	53.32%
			Positive	91	11.64%

In contrast, neutral emotions dominate in Theme 4 “Postpartum recovery” and Theme 5 “Caesarean section surgical experience,” where many posts describe experiences such as wound care, limited mobility and breastfeeding difficulties in an informational, narrative tone. Theme 3 “Pregnancy diaries and mother–baby experience sharing” presents a relatively balanced emotional structure, encompassing both anxiety about pregnancy and childbirth risks and parenting pressures, as well as joy and satisfaction at the arrival of new life.

### 4.4 Performance of the sentiment classification model

The performance of the BERT-based sentiment classifier was evaluated on a held-out test set. The model achieved a high overall accuracy and F1-score, and performed well in distinguishing negative posts from non-negative ones. The macro-average area under the ROC curve (AUC-ROC) was approximately 0.89, indicating strong discriminative ability across different decision thresholds. These results suggest that the proposed deep learning model can reliably identify childbirth-related sentiments in Chinese social media texts, providing a solid technical foundation for subsequent analyses based on sentiment distribution.

## 5 Discussion

### 5.1 The “bittersweet” pattern of online childbirth narratives and differences between vaginal birth and caesarean section

The combined LDA–BERT results reveal a distinct “bittersweet” pattern in online childbirth narratives. On the one hand, negative emotions are more prevalent than positive ones at the corpus level, especially in posts describing the vaginal birth process. This is consistent with the intense pain, physical exhaustion and perceived loss of control commonly reported during labour. Women frequently depict themselves “on the edge of collapse,” “screaming from the pain” or “feeling completely out of control,” which highlights the psychological burden and potential risk of traumatic birth experiences.

On the other hand, the theme of “emotional value and family meaning of childbirth” shows that painful and even traumatic experiences are often reinterpreted, with time, as meaningful sacrifices for the child and family. Many women describe how they “would still choose to have this baby despite everything,” or how witnessing the baby’s first cry and the partner’s tears transformed the experience into a source of pride, gratitude and strengthened family bonds. This indicates that meaning-making and narrative reconstruction can partially alleviate the negative impact of childbirth and facilitate psychological adaptation.

Narratives of caesarean section differ in tone. At the surface level, caesarean section posts appear more neutral and less emotionally intense, with many women emphasizing controllability and predictability (e.g., “no need to endure long contractions”). Yet, a closer look reveals hidden tensions: some express guilt or ambivalence about “choosing the easier way,” worry about long-term health implications, or feel regret for not having experienced vaginal birth. Thus, while caesarean section may reduce acute uncertainty, it introduces other psychosocial contradictions that need to be addressed in childbirth education and counseling.

### 5.2 The role of social media in informational and emotional support

The strong presence of themes such as “pregnancy diaries and mother–baby experience sharing” underscores the central role of social media in providing both informational and emotional support for women around childbirth. Many posts explicitly aim to “leave a record” or “help later mothers,” offering practical tips (e.g., pain management strategies, what to pack for hospital, breastfeeding positions) and sharing “lessons learned” from complications or unexpected events. This peer-to-peer information flow complements formal medical guidance, especially in areas where hospital-based education is fragmented or time-limited.

At the emotional level, comment sections and repost interactions often function as informal support communities. Women receive empathy, encouragement and validation from strangers who have had similar experiences. Expressions such as “you are so brave,” “I was exactly like this” or “thank you for making me less scared” illustrate how social media mitigates loneliness and normalizes intense feelings during childbirth. For women preparing to give birth, reading others’ experiences can reduce anxiety by providing vicarious exposure, although in some cases highly negative or sensational stories may also amplify fear and risk perception.

At the same time, the findings also hint at the ambivalence of social media as a support space. The algorithmic amplification of emotionally extreme content may skew perceptions of typical childbirth experiences, and user-generated advice sometimes conflicts with evidence-based medical recommendations. This implies that health professionals cannot simply ignore social media, but need to understand and, where possible, engage with these online narratives, in order to correct misinformation, respond to common concerns and build more constructive channels of communication.

### 5.3 Implications for clinical practice and public health

The findings provide data-driven, actionable directions for optimizing maternal health services, particularly when viewed through a cross-cultural lens. The “bittersweet” pattern identified in this study shares similarities with non-Chinese cultural contexts. For instance, studies on Australian and Swedish women have also found that intense memories of pain frequently coexist with the joy of new motherhood, suggesting a cross-cultural universality in meaning-making (29, 30). However, the emotional distribution of caesarean sections differs from Western findings. While Swedish studies often characterize postoperative experiences as predominantly pain-oriented and linked to a sense of “failing to give birth naturally” (30), the Chinese narratives present a more balanced emotional profile. In the Chinese context, caesarean section is sometimes reconstructed as an active and controllable “smart choice” (31). This divergence reflects differing socio-cultural attitudes toward bodily autonomy and medical interventions, which must be considered when designing targeted public health strategies.

First, interventions addressing the highly negative emotions during vaginal birth should focus on structured pain management and humanized care. The intense narratives regarding contraction pain and the perceived “invalid waiting time” for epidurals highlight the urgent need to expand the accessibility of labour analgesia (32). Hospitals should optimize anesthesiology resources to reduce waiting times. Additionally, the reported feelings of helplessness and loss of dignity during procedures underscore the necessity of continuous emotional support, such as doula companionship, to help women regain a sense of control (33).

Second, the unique cultural framing of caesarean sections calls for nuanced interventions. While some young mothers view caesarean delivery as a “smart choice” for its predictability (31), their narratives often overlook long-term health risks. Public health policies should respect women’s autonomy while ensuring comprehensive education on both the short-term benefits and long-term consequences of caesarean sections. Furthermore, standardized postoperative pain management protocols must be enhanced.

Third, from a digital health perspective, health authorities can leverage social media’s positive potential by establishing official accounts to share evidence-based, empowering birth stories. This can help balance the disproportionate spread of “fear-inducing narratives” online. The analytical framework developed in this study can also be deployed as a real-time monitoring tool to identify maternal psychological crises and unmet health needs.

## 6 Conclusion and future work

This study conducted a multidimensional analysis of Chinese women’s childbirth experiences on social media by integrating LDA topic modeling with BERT-based sentiment analysis. Using a corpus of over 17,000 childbirth-related posts from Weibo and REDnote, we identified six major themes that together portray a rich landscape of childbirth narratives, ranging from high-risk pregnancy and labour processes to postoperative recovery, family meaning and daily mother–baby diaries. Sentiment analysis revealed a pronounced “bittersweet” pattern, with negative emotions more prevalent overall, particularly in vaginal birth narratives, yet interwoven with positive feelings of love, gratitude and life meaning. Vaginal birth and caesarean section narratives exhibited distinct emotional profiles and psychosocial tensions, highlighting the need for differentiated clinical communication and support.

Methodologically, the study demonstrates the feasibility and value of combining LDA and deep learning–based sentiment analysis for mining large-scale, user-generated health narratives in Chinese. The integrated framework not only captures the thematic structure of childbirth stories, but also quantifies emotional patterns across topics, thereby offering a scalable tool for obstetric humanities research and health communication studies.

However, several limitations must be acknowledged, which point toward directions for future work. First, the data were drawn exclusively from Weibo and REDnote, which exhibit selection bias. These platforms are predominantly used by younger, highly educated, and digitally literate women residing in urban areas (34, 35). Consequently, the sample may over-represent this demographic while underrepresenting the childbirth experiences of women in rural areas, those with lower educational attainment, or the digitally marginalized. Second, this study relies on observational social media texts, which reflect women’s self-presentation in public digital spaces rather than direct clinical measurements. These narratives are subject to presentation biases influenced by platform norms and audience expectations (36). Therefore, caution must be exercised when linking narrative patterns to actual psychological or clinical outcomes (e.g., clinical diagnoses of PTSD or PPD). Third, the keyword-based search strategy may inherently favor users who are willing and able to articulate detailed stories (e.g., “diaries”), potentially missing those who express their experiences differently. Finally, the lack of clinical background information (e.g., parity, specific obstetric comorbidities) restricts deeper clinical interpretation of the findings. Future research should consider longitudinal and mixed-methods approaches, or link social media data with electronic health records, to reveal the dynamic processes of coping and to achieve a more comprehensive understanding of childbirth experiences.

## Data Availability

The original contributions presented in the study are included in the article/supplementary material, further inquiries can be directed to the corresponding author.
